# Fibronectin Matrix Assembly Suppresses Dispersal of Glioblastoma Cells

**DOI:** 10.1371/journal.pone.0024810

**Published:** 2011-09-30

**Authors:** Joshua Sabari, Daniel Lax, Daniel Connors, Ian Brotman, Eric Mindrebo, Christine Butler, Ildiko Entersz, Dongxuan Jia, Ramsey A. Foty

**Affiliations:** Department of Surgery, UMDNJ-Robert Wood Johnson Medical School, New Brunswick, New Jersey, United States of America; Dana-Farber Cancer Institute, United States of America

## Abstract

Glioblastoma (GBM), the most aggressive and most common form of primary brain tumor, has a median survival of 12–15 months. Surgical excision, radiation and chemotherapy are rarely curative since tumor cells broadly disperse within the brain. Preventing dispersal could be of therapeutic benefit. Previous studies have reported that increased cell-cell cohesion can markedly reduce invasion by discouraging cell detachment from the tumor mass. We have previously reported that α5β1 integrin-fibronectin interaction is a powerful mediator of indirect cell-cell cohesion and that the process of fibronectin matrix assembly (FNMA) is crucial to establishing strong bonds between cells in 3D tumor-like spheroids. Here, we explore a potential role for FNMA in preventing dispersal of GBM cells from a tumor-like mass. Using a series of GBM-derived cell lines we developed an in vitro assay to measure the dispersal velocity of aggregates on a solid substrate. Despite their similar pathologic grade, aggregates from these lines spread at markedly different rates. Spreading velocity is inversely proportional to capacity for FNMA and restoring FNMA in GBM cells markedly reduces spreading velocity by keeping cells more connected. Blocking FNMA using the 70 KDa fibronectin fragment in FNMA-restored cells rescues spreading velocity, establishing a functional role for FNMA in mediating dispersal. Collectively, the data support a functional causation between restoration of FNMA and decreased dispersal velocity. This is a first demonstration that FNMA can play a suppressive role in GBM dispersal.

## Introduction

Glioblastoma (GBM) is the most common and most malignant primary brain tumor with a median survival of 12-months [1], [2]. GBM represents 60% of all gliomas and 30% of all intracranial tumors [3]. The hallmark of these aggressive tumors is their ability to rapidly disperse into surrounding brain tissue. Despite aggressive surgical intervention, directed radiation therapy, and systemic chemotherapy, median survival remains poor. Recent data from US and European cancer registries report median survival rates in GBM patients of less than 30% at one year, 5% at three years, and 3% at five years [4]. Good prognostic factors include young age at diagnosis (<50 years old), initial Karnofsky Performance Score >80, the extent of resection [5], and low Survivin and Ki-67 expression [6]. Hypermethylation of the MGMT promoter is also generally considered to correlate with good prognosis [7] and a favorable response to chemotherapy by alkylating agents [8].

GBM is lethal partly due to its propensity for early microscopic dispersal prior to diagnosis, confounded by the practical improbability of a complete resection including all microscopic tumor remnants. Accordingly, gliomas almost always recur, and in 90% of patients, recurrence is often within 2 to 3 cm from the border of the original lesion [9]. This local dispersal generally leads to very poor clinical outcome [10]. Thus, preventing tumor cell dispersal would be of significant therapeutic benefit.

Cytokines [11], lymphokines [12], [13], various adhesion molecules [14], growth factors[15], metalloproteases [16], [17], calcium-activated proteases such as calpain-2 [18], and a myriad of extracellular matrix components [14] have been identified as potential regulators of brain tumor invasion. However, no single factor can be regarded as a “master switch,” and the process is likely a dynamic interplay between multiple components. Therefore, a more global parameter of brain tumor behavior is necessary to understand the effects of various contributors to the equilibrium between the invasive and noninvasive states. A critical component of this equilibrium is the ability of tumor cells to remain adherent to one another, rather than to invade and infiltrate the surrounding tissue. We have previously reported that increased cell-cell cohesion can markedly reduce invasion in several cancer models including human lung cancer [19], fibrosarcoma [20], and glioma cell lines [21]. Thus, increasing cell-cell cohesion could, in principle, represent a possible strategy of discouraging detachment of cells from the tumor mass and subsequent dispersal.

Cell-cell cohesion can be regulated by several cell surface adhesion molecules, including cadherins and integrins. Cadherins are a large family of calcium-dependent cell surface adhesion molecules and are thought to be the principal mediators of direct intercellular cohesion [22]. Cadherins have been linked with transition to malignancy of a variety of tumors. Most notably, E-cadherin has been implicated as being an important molecule in invasion and metastasis of carcinoma, largely because its expression is in some cases inversely correlated with tumor aggressiveness [23]. However, a general survey of the literature indicates that downregulation of cadherin expression is not always associated with increased invasiveness. For example, cadherin-dependent adhesion, rather than discouraging cell migration, appears to promote cell adhesion, neurite outgrowth and overall migratory capacity of human U373-MG glioblastoma cells [24]. Moreover, even if cadherin expression could be manipulated to increase cohesion in GBM, this method would be cumbersome, requiring up-regulation of cadherin expression by an in vivo gene-transfer strategy. Ideally, in the best interest of safety and efficacy, upregulation of cell-cell cohesion in GBM should be induced pharmacologically.

The synthetic glucocortocoid dexamethasone (Dex), has been demonstrated to reactivate the ability of cells to assemble fibronectin into a complex fibrous matrix through a process termed fibronectin matrix assembly (FNMA) [25], [26]. We have previously shown that α5β1-fibronectin interaction can mediate strong intercellular cohesion of CHO cells when grown as three-dimensional aggregates [27] and that this extracellular matrix-mediated cohesion is largely dependent on the ability of cells to assemble fibronectin into a matrix [28]. FNMA has been extensively studied in many cancer models, however, relatively little is known in regard to its potential role in glioblastomas. A recent study reported that malignant gliomas demonstrate increased staining of fibronectin matrix that distinguish them from normal brain tissue and that interfering with matrix assembly can render tumors more susceptible to chemotherapy, suggesting a role for FNMA in brain tumor biology [29]. We previously showed that Dex increases cohesion and decreases invasion of glioblastoma cells [21]. We therefore reasoned that Dex treatment may represent a potential strategy for modulating FNMA in glioblastoma cells and that, in principle, this should decrease the rate at which tumor cells detach from and escape the tumor mass. We exploited the fact that glioblastoma cells form three-dimensional spheres when placed in hanging drop culture. We then developed a method of quantifying the spreading velocity of these aggregates when plated onto tissue culture plastic, in much the same way as a “wetting” phenomenon observed for ordinary liquids as they interact with a substrate [30]. We also explored the relationship between spreading velocity and FNMA.

## Materials and Methods

### Cell lines

The malignant astrocytoma cell lines U87-MG, U118-MG and LN-229 were obtained from the American Type Culture Collection (ATCC, Manassas, VA). U87-MG cells were maintained in Eagle's Minimum Essential Medium (ATCC, Manassas, VA) containing 10% fetal bovine serum (FBS, Hyclone, Logan, UT), 2 mM L-glutamine, 1 mM sodium pyruvate, 0.1 mM nonessential amino acids, and 1.5 g/L sodium bicarbonate (Gibo-BRL, NY). U118-MG and LN-229 cells were maintained in Dulbecco's Modified Eagle's Medium (Gibco-BRL, NY) containing 10% and 5% fetal bovine serum, respectively, 4 mM L-glutamine, 4.5 g/l glucose, and 1.5 g/l sodium bicarbonate. All media were supplemented with 100 µg/ml streptomycin sulfate, 100 U/ml penicillin G sodium, 0.25 µg/ml amphotericin B, and 50 µg/ml gentamicin sulfate (Gibco-BRL, NY). All cell lines were maintained at 37°C, 95% humidity, and 5% CO_2_.

### Aggregate preparation

Near-confluent cell monolayers were dissociated from 10-cm tissue culture plates with 0.05% trypsin/0.53 mM EDTA (TE). Dispersed cells were washed in complete medium to inhibit the trypsin then centrifuged to pellet the cells. Pellets were washed with PBS and resuspended in complete medium to a concentration of 1×10^6^ cells/ml. Ten-µl aliquots were deposited on the underside of the lid of a 10-cm tissue culture dish. The bottom of the dish contained 5-ml of PBS and served to prevent evaporation of the drops by forming a hydration chamber. Hanging drops were created by inverting the lid over the hydration chamber. The drops were incubated at 37°C, 95% humidity, and 5% CO2 for 24–48 hours, allowing cells to coalesce and form spherical aggregates.

### Measurement of aggregate dispersal velocity

A single aggregate was plated into a 35 mm tissue culture plate containing pre-warmed CO_2_-independent medium with 10% FBS. The plate was then transferred to a heated microscope stage of a Nikon Eclipse TE-300 microscope equipped with a Photometrics Coolsnap ES B&W digital camera and connected to an Apple G4 computer running IPLab imaging software (Exton, PA). Aggregates were allowed to adhere to the plates for 15 minutes, whereupon images were captured every 5 minutes for up to 8 hours. Images were analyzed using ImageJ software. Aggregate diameter was measured at 4 different points and averaged. Diameter measured at t_0_ was used to normalize each subsequent image time-point. The normalized diameter was then plotted as a function of time. The relationship between aggregate diameter and time was analyzed by linear regression. Regression lines for which the correlation coefficient, r^2^, was greater than 0.95 were used to calculate dispersal velocity from the slope. Between 10 and 15 aggregates were used to generate each data set.

### Treatment of cells with FNMA-inducing agents

Fifty-thousand cells were plated into wells of a 24-well tissue culture plate and incubated in complete medium until cells reached 80% confluence. Adhering cells were then washed once with PBS then incubated in medium containing either 10^−6^ M Dex (Sigma, St. Louis, MO), 25 µM MEK inhibitor (MEKi) PD 98059 (Calbiochem, Gibbstown, NJ), or 0.5 µM Geldanamycin (GA, Calbiochem, Gibbstown, NJ) for 24 hours. For carrier controls, cells were incubated in either sterile distilled H_2_O (for Dex) or DMSO (for MEKi and GA). Control and treated cells were either utilized to generate aggregates for measurement of dispersal velocity as described above, or were assessed for their ability to assemble a fibronectin matrix.

### Assessment of FNMA by immunofluorescence

Adherent cells were blocked in CAS-Block (Invitrogen, Carlsbad, CA) for 30 minutes. Fibronectin matrix was detected by incubating cells in anti-FN antibody (ab6584, Abcam, Cambridge, MA) for 1 hour at room temperature (RT). After 3 washes with HBSS cells were incubated in Alexafluor-568 secondary antibody (Invitrogen, Carlsbad, CA) for 30 minutes. After several washes in HBSS, cells were incubated in Syto-16 nuclear stain (Invitrogen, Carlsbad, CA) for 15 minutes and mounted in FluorSave reagent (Calbiochem, Gibbstwon, NJ). Cells were visualized using fluorescence microscopy on a Nikon Eclipse TE300 inverted microscope. Images from red and green channels were captured using a Photometrics Coolsnap ES digital camera connected to an Apple G4 computer, assigned false color, and merged in IPLab imaging software.

### Aggregate compaction assay

Cells were trypsinized, washed, counted using a BioRad TC10 automated cell counter, and resuspended in complete tissue culture medium at a concentration of 2.5×10^6^ cells/ml. Ten µl hanging drops were generated as described above and incubated for 24 hours. Within this time-frame, cells coalescing at the bottom of the hanging drops formed sheets. Images were captured and analyzed using IP Lab imaging software. Each image was adjusted for optimum contrast. Outlines were automatically traced and the number of pixels within the outlines were calculated. Data points representing the mean and standard error for aggregate surface area expressed in pixels (a measure of aggregate compaction) were calculated from 8–10 aggregates of each cell line.

### Assessment of α5 integrin, pan-cadherin expression and fibronectin secretion by immunoblot analysis

To assess expression of **α5**-integrin, 25 µg of protein was separated on a 7% SDS-PAGE gel under non-reducing conditions and blotted to PVDF membranes using standard protocols. Blots were blocked in Blotto (5% nonfat dry milk, 0.05% Tween-20, in PBS) for 1 h, then incubated at 4°C overnight in Blotto with 10 µg/ml rabbit polyclonal antibody against α5 integrin (Millipore, Billerica, MA). For pan-cadherin and actin, protein was separated under reducing conditions and blots were blocked and incubated in pan-cadherin (CH-19, Sigma, MO) or actin antibody (Sigma, ST. Louis, MO). Blots were rinsed three times in TBS-0.2% Tween-20, and incubated at room temperature for 1 h in either anti-rabbit or anti-mouse IgG-HRP (Thermo Scientific, Waltham, MA). After three more rinses, blots were developed using enhanced chemiluminescence and exposed to X-ray film. Films were scanned using an HP ScanJet 8300 digital scanner and digital images were analysed using the gel plotting function in ImageJ. The optical densities (OD) of the bands representing pan-cadherin expression and their corresponding actin bands were quantified from three separate experiments. Cadherin expression was normalized by expressing OD of the cadherin signal by that of the corresponding actin signal. Mean OD was compared by ANOVA and Tukey's Multiple Comparisons Test (MCT).

To assess FN secretion, cells were plated at equal densities in complete medium containing FN-depleted FBS and either carrier or FNMA-inducing agents as described above. Serum was depleted of FN as described previously [31]. After 24 h, cells and media were collected together, and cells were pelleted by centrifugation. One hundred µl of tissue culture medium from each sample was mixed with 100 µl of gelatin-sepharose beads (GE Healthcare, Piscataway, NJ). Beads and media were rotated for 30 min at room temperature (RT) then washed five times in ice-cold PBS, followed by boiling in SDS sample buffer containing 5% β-mercaptoethanol. Samples were analyzed by SDS-PAGE, followed by immunoblotting with a biotin-conjugated anti-FN antibody (AB6584, abcam, Cambridge, MA) and streptavidin-HRP (Pierce, Rockford, IL). Blots were developed as described above.

### Assessment of surface α5β1 integrin expression by flow cytometry

Cells were detached from near-confluent tissue culture plates with trypsin/EDTA (TE; Gibco-BRL, NY), washed three times with ice-cold Hanks' balanced salt solution (HBSS), and resuspended in HBSS at a concentration of 1×10^7^ cells/ml. One hundred µls were aliquoted in duplicate into centrifuge tubes. Five µg/ml of anti-integrin antibody (**α5**/FnR mouse monoclonal P1D6, Calbiochem, Gibbstown, NJ) was added to one of the duplicates and tubes were incubated on ice for 30 minutes with agitation. After two washes with HBSS, cells were re-suspended in ice-cold HBSS at 1∶100 dilution of Alexa-Fluor 488-conjugated goat-anti-mouse IgG secondary antibody (Invitrogen, Carlsbad, CA). After 30 minutes, cells were washed twice with HBSS and analyzed using a Becton-Dickinson FacsCalibur flow cytometer. Secondary antibody only controls were used to establish gating parameters.

### Blocking of FNMA by the 70 KDa fragment of fibronectin

To assess the effects of the 70 KDa fragment on FNMA by immunofluorescence, cells were plated into 24-well tissue culture plates and incubated with 50 µg/ml 70 KDa fibronectin fragment (Sigma, MO). Matrix was detected as described above. For hanging drop cultures, cells were suspended at a concentration of 1×10^6^ cells/ml and 10 µl hanging drops containing 50 µg/ml 70 KDa fragment were formed as described above. Hanging drops were incubated for 24–48 hours. These aggregates were used for spreading velocity assays. For compaction assays, cells were suspended at a concentration of 2.5×10^6^ cells/ml in 70 KDa-containing medium and hanging drops were incubated for 24 hours.

### Assessment of FNMA by differential solubilization assay

The assembly of high molecular weight FN multimers (HMWFM) was assessed using a deoxycholic acid (DOC) differential solubilization protocol as previously described [28], [32]. Briefly, 3D aggregates were generated by the hanging drop method either in the absence and/or presence of Dex and the 70 KDa fragment. After 24 h of incubation, aggregates were lysed in a DOC lysis buffer (2% sodium deoxycholate, 0.02 M Tris-HCl, pH 8.8, 2 mM PMSF, 2 mM EDTA, 2 mM iodoacetic acid, and 2 mM *N*-ethylmaleimide), passed through a 26 gauge needle, and centrifuged at 15×G for 20 minutes at 4°C. The supernatant containing the DOC soluble fraction was transferred to a fresh tube. The pellet from the 20-minute spin, representing the DOC-insoluble fraction, was solubilized using SDS lysis buffer (1% SDS, 25 mM Tris-HCl, pH 8.0, 2 mM PMSF, 2 mM EDTA, 2 mM iodoacetic acid, and 2 mM *N*-ethylmaleimide). The insoluble fraction was separated by SDS –PAGE under non-reducing conditions, the soluble fraction under reducing conditions. Protein was transferred to PVDF and probed with an anti-FN antibody (ab6584, Abcam, UK). Blots containing the soluble fraction were also probed with an anti-actin antibody (A2066, Sigma, St. Louis, MO) to control for equal loading.

### Statistical analysis

Linear regression analysis was used to describe the relationship between normalized aggregate diameter and time, for the effect of Dex-treatment on the slope of the velocity curve, and for the relationship between velocity and aggregate size. In each case, a correlation coefficient was generated, a Run's test was used to determine departure from linearity, and an F test was used to determine whether the slopes differed significantly. Ten-fifteen aggregates from each cell line were used to generate the regression lines. ANOVA and Tukey's MCT were used to determine whether significant differences exist i) in dispersal velocity between the three cell lines, ii) in aggregate size in response to drug treatment, (iii) to compare the effect of drug treatment and presence of the 70 KDa fragment on spreading velocity, and (iv) to compare OD of the cadherin and actin bands in the pan-cadherin western blot. A p-value<0.05 was considered significant.

## Results

### Aggregate dispersal velocity assay

**Figure 1:**
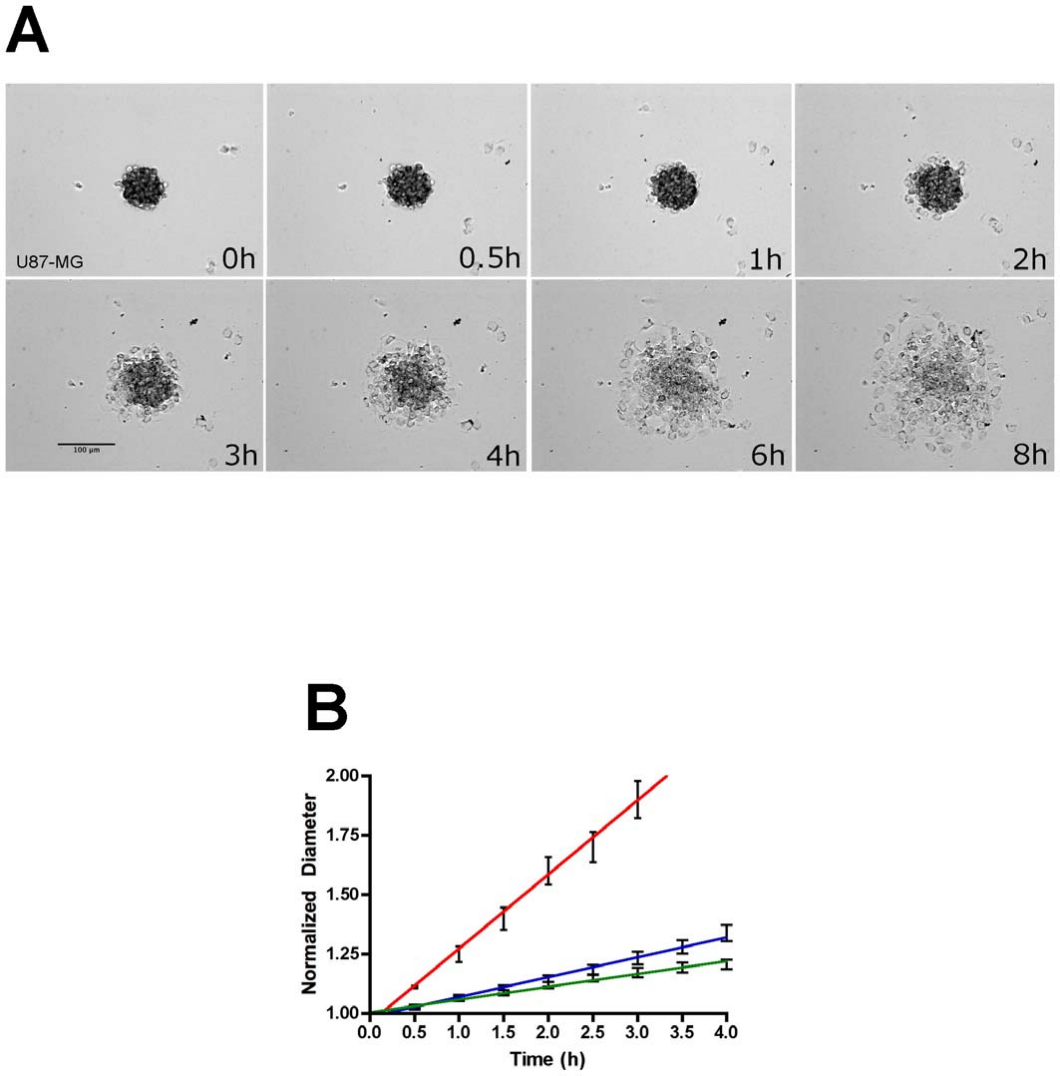
Time-dependent dispersal of aggregates of U87-MG cells. A) Aggregates of U87-MG cells approximately 50 µm in diameter, plated onto tissue culture plastic and incubated in complete medium under standard tissue culture conditions, spread to a monolayer within 8 hours. This dispersal can be quantified as spreading velocity by calculating the slope of the line representing the change in normalized aggregate diameter over time. A time-lapse movie showing aggregate dispersal can be viewed in Movie S1. B) Linear regression analysis of aggregate spreading. The relationship between normalized aggregate diameter and time was found to be linear for aggregates of all three-cell lines. The red line represents aggregates of U87-MG cells, whereas the blue and green lines represent LN-229 and U118-MG, respectively. Ten to 15 aggregates were used to calculate the mean normalized diameters at each time-point. Standard deviation bars are depicted.

Aggregates of U87-MG, LN-229, and U118-MG cells, when plated on tissue culture plastic in complete medium, adhere and spread to a near-monolayer within 8 hours (Fig. 1A). This dispersal behavior was quantified by measuring the change in diameter over time. When aggregate diameter was plotted as a function of time, the relationship appeared to be linear. This was confirmed by linear regression analysis. As can be seen in Fig. 1B, the correlation coefficient, r^2^, for U87-MG, LN-229, and U118-MG was 0.995, 0.990, and 0.989, respectively, indicating a high probability for linearity. This was further confirmed by testing for departure from linearity by Run's test, which showed that the lines were not significantly non-linear (P = 0.78, 0.07, and 0.07 for U-87MG, LN-229, and U-118MG, respectively). The difference between the slopes was also analyzed by F test and found to be significant between the 3 cell lines (P<0.0001). We also tested whether aggregate size correlates with dispersal velocity. Velocity and size data from all cell lines was combined and subjected to regression analysis. Fig. S1 shows that the slope of the regression line is essentially zero (r^2^ = 0.013), indicating that there is no relationship between aggregate diameter and spreading velocity. Based on these data, it was possible to calculate aggregate dispersal velocity from the slope of the regression line. Since dispersal velocity must also be influenced by cell motility, we asked whether these cell lines differed in their migration rates. We performed assays in which equal numbers of cells were plated onto filters containing 8 µm pores and migrating cells counted after 16 hours in tissue culture. As can be seen in Fig. S2, all three-cell lines migrated at the same rate.

### Dispersal velocity of aggregates of U87-MG, LN-229, and U118-MG

**Figure 2:**
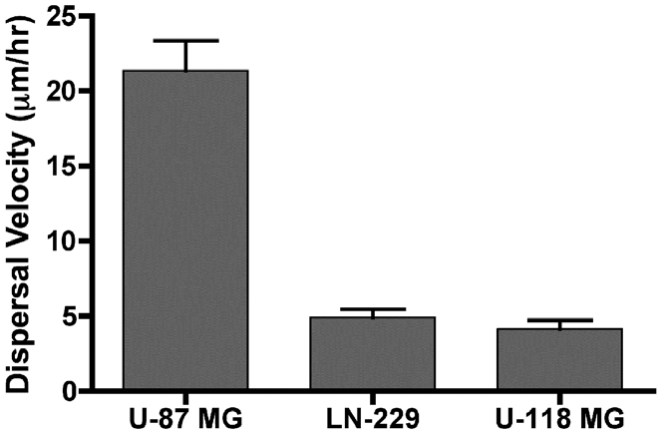
Aggregate dispersal velocity of 3-glioblastoma cell lines. This bar graph depicts the spreading velocities of aggregates of U87-MG, LN-229, and U118-MG cells. Data sets included sample sizes of 14, 10, and 11 aggregates, respectively. Aggregate spreading was monitored over an 8-hour period. Movies of the spreading were captured and images at various time points were analyzed. Normalized aggregate diameter was plotted as a function of time, generating a slope, which was used to calculate spreading velocity. As can be seen here, the spreading velocity of aggregates of U87-MG cells is considerably faster than that of either LN-229 or U118-MG (ANOVA, p<0.0001 and Tukey's MCT).

As can be seen in Fig. 2, the dispersal velocity of U87-MG cells is 21.4±2.9 µm/h whereas that of LN-229 and U118-MG is lower, at 4.9±0.6 µm/h and 4.1±0.6 µm/h, respectively. ANOVA and Tukey's MCT demonstrated statistically significant differences between the three cell lines (P<0.0001) and specifically between U87-MG and both LN-229 and U118-MG (P<0.001) but not between LN-229 and U118-MG (P>0.05). A potential molecular mechanism underlying the difference in dispersal velocity was then explored.

### Expression of potential regulators of aggregate dispersal velocity

**Figure 3:**
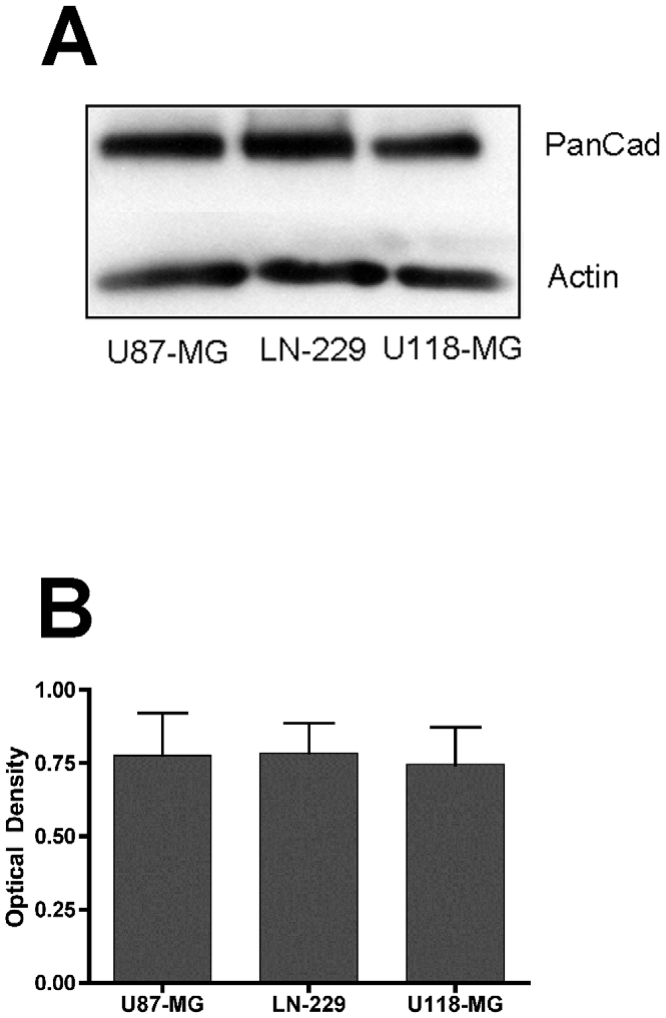
Assessment of pan-cadherin expression by glioma cells. A) Overall cadherin expression by the three cell lines was assessed by immunoblot analysis using a pan-cadherin antibody (clone CH-19, Sigma). Bands, corresponding to the molecular weight of cadherin, are evident. Actin was used as a loading control. B) Quantification of cadherin expression by densitometric analysis. Optical density (OD) of cadherin bands was measured in triplicate as described in the Methods section. These measurements were then normalized by expressing OD of the cadherin signal by that of the corresponding actin signal. Here, the average normalized cadherin OD of the three cell lines is compared. No difference in cadherin expression was detected (ANOVA, p>0.05).

**Figure 4:**
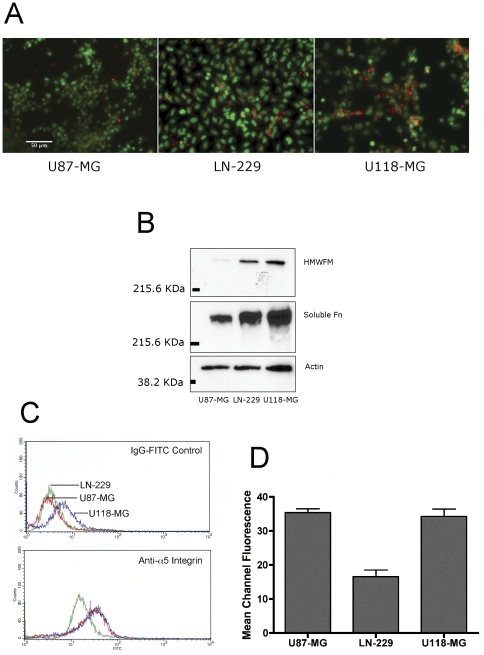
Assessment of fibronectin matrix assembly and α5 integrin expression by glioma cells. A) Immunofluorescence analysis of FNMA by glioma cells. Cells were plated at equal density and incubated for 24 hours, whereupon they were stained with an anti-fibronectin antibody and an Alexa-568-conjugated secondary IgG. Cell nuclei were counterstained using Syto-16 live-cell nuclear stain. Images from green and red channels were captured, pseudo-colored, and merged in IP lab. Nuclei are depicted in green and fibronectin matrix is depicted in red. B) Biochemical analysis of FNMA by differential solubilization assay and immunoblot analysis. The assembly of high molecular weight fibronectin multimers (HMWFM) by U87-MG, LN-229, and U118-MG cells was assessed using DOC differential solubilization assay. DOC-soluble and insoluble fractions were separated by SDS-PAGE and analyzed by immunoblotting using an anti-fibronectin antibody. Actin in the soluble fraction was used as loading control. C) Analysis of cell surface α5β1 integrin expression by flow cytometry. Cells were trypsinized from near-confluent tissue culture plates, washed, and incubated with a mAb specific for the extracellular domain of α5 integrin, followed by incubation with a FITC-conjugated secondary antibody. The control panel (left) represents cells incubated with secondary antibody only. The right panel represents cell surface expression of α5 integrin. D) Comparison of α5β1 integrin expression by measuring mean fluorescence intensity (MFI). Cells were incubated in either IgG-FITC or in anti-α5β1 integrin antibody and IgG-FITC as described in Materials and Methods. Cells were appropriately gated and statistical analyzed using CellQuest software to calculate MFI values for controls and their matched samples. The MFI values of controls were subtracted from the MFI values of their matched samples, yielding a net MFI value for each cell line. Five separate experiments were performed using exact cytometer settings. The MFI data were averaged and mean MFI were compared by t-test or ANOVA and Tukey's MCT. MFI of U87-MG and U118-MG were statistically identical (ANOVA, P<0.0001, Tukey's MCT, p>0.05). MFI of LN229 was lower than that of either U87-MG or U118-MG (ANOVA, p<0.0001, Tukey's MCT, p<0.001).

Previous studies have shown that aggregate dispersal is a competitive outcome between cell-cell cohesion and cell-substratum adhesion [33]. In our model, the substrate is kept constant. Accordingly, differences in dispersal velocity must be attributable to differences in cell-cell cohesion. We therefore asked whether these three cell lines expressed different levels of cell surface adhesion proteins that could account for differences in their dispersal velocities. A possible difference in cadherin expression was assessed by immunoblot analysis using a pan-cadherin antibody. Fig. 3A shows that cadherin expression relative to actin is similar for all three cell lines. We then quantified optical density of the cadherin bands normalized to actin and compared the normalized data from three experiments by ANOVA and Tukey's MCT. No difference in expression was detected (Fig. 3B). α5β1 integrin-fibronectin interaction [27] and it's regulation of FNMA [28] has previously been shown to mediate indirect cell-cell cohesion in 3D aggregates. Accordingly, these three cell lines were assessed for their ability to assemble fibronectin into a matrix. As can be seen in Fig. 4A, high dispersal velocity U87-MG cells appear to lack the ability to assemble a matrix as compared with the slower-dispersing LN-229 and U118-MG cell lines. This was confirmed by immunoblot analysis. Using a DOC differential solubilization assay, we show that U87-MG cells are deficient in the expression of high molecular weight fibronectin multimers as compared to LN-229 and U118-MG cells. Moreover, there also appears to be a difference in the presence of fibronectin in the soluble fraction. This likely represents a difference in their capacity to bind fibronectin at the cell surface (Fig. 4B). This difference in capacity for FNMA was not due to differences in α5β1 integrin expression, since cell lines expressed similar levels as demonstrated by significant overlap of histograms as in flow cytometry experiments (Fig. 4C). This was confirmed by comparing mean fluorescence intensity (MFI) of the normalized histogram data for U87-MG, LN229 and U118-MG cells. As can be seen in Fig. 4D, MFI values of five separate experiments are similar for U87-MG and U118-MG cells, suggesting that these lines express similar levels of α5β1 integrin. MFI values of LN229 cells are lower, indicating that α5β1 integrin expression by the three cell lines is variable.

### FNMA can be regulated by drug treatment and can influence aggregate compaction

**Figure 5:**
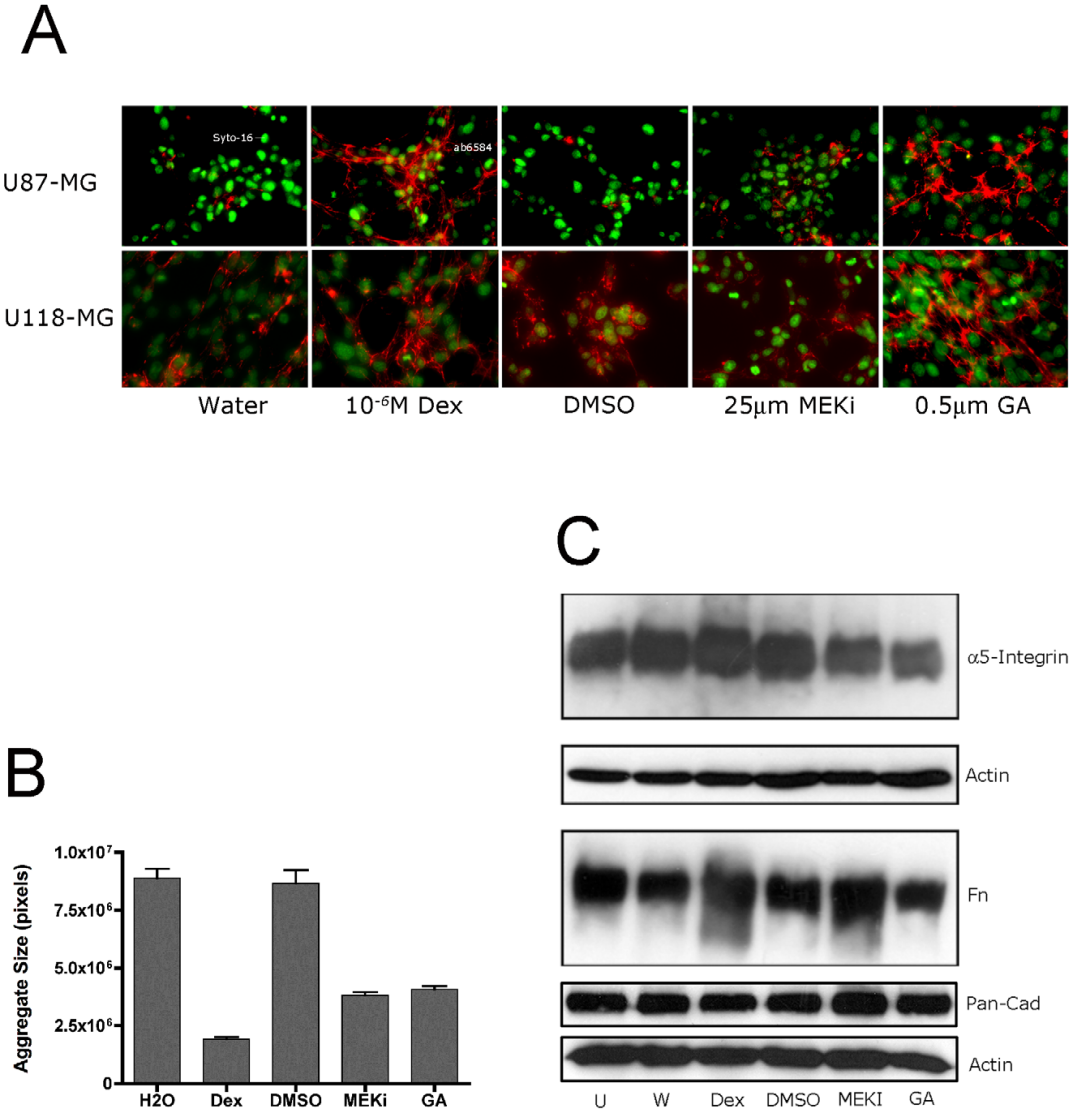
Reastoration of FNMA promotes compaction of glioma cells. A) Restoration of FNMA by Dexamethasone, MEK inhibitor, and Geldanamycin. Cells were plated at equal densities and incubated for 24 hours in the presence of Dexamethasone, MEK inhibitor, Geldanamycin, or carrier-only controls. FNMA by U87-MG and U118-MG was assessed by immunofluorescence as previously described. Fibronectin matrix was detected using anti-fibronectin antibody (ab6584) and Alexa-568-conjugated secondary IgG and is depicted in red. Nuclei were stained with Syto-16 live-cell nuclear stain and are depicted in green. Dex and GA appear to be most effective in restoring FNMA in both lines. B) Restoration of FNMA results in aggregate compaction. Untreated and drug-treated U87-MG cells were placed in hanging drop culture and incubated for 24 hours, whereupon aggregate size was measured as previously described. Depicted are mean aggregate size in pixels, with corresponding standard error. Note the marked compaction of aggregates treated with Dex, MEKi, or GA relative to carrier controls (water and DMSO). C) Immunoblot analysis of α5 integrin and pan-cadherin expression and of FN secretion in response to drug treatment. Antibodies specific for α5 integrin, secreted fibronectin, or pan-cadherin were used to determine whether drug treatment altered expression or secretion levels. Immunoblot analysis reveals that drug treatment does not appear to upregulate the expression of either α5 integrin nor of any cadherin recognized by the CH-19 pan-cadherin antibody. FN secretion also appears to be unaffected by drug treatment. Depicted, are protein levels relative to actin, which was used as loading control.

Gliomas are known to exhibit high Ras activity [34] and this has been shown to impede FNMA [35]. We asked whether restoring FNMA would decrease dispersal velocity. This was accomplished by treating U87-MG and U118-MG cells with drugs previously demonstrated to restore FNMA, then assessing influence on aggregate compaction and dispersal velocity. Fig. 5A shows that for U87-MG cells, Dex and GA significantly increased fibronectin matrix as compared to controls. A different effect was observed when U118-MG cells were treated. In that case, only GA appeared to significantly upregulate FNMA. In previous studies, we showed that restoring FNMA in CHO cells resulted in increased compaction and aggregate cohesion [27]. We therefore asked whether drug treatment and the attendant increase in fibronectin matrix influence compaction of these brain tumor cell lines. Fig. 5B represents a compaction assay for control and drug-treated U87-MG cells. Analysis by ANOVA and Tukey's MCT shows that drug treatment significantly decreased aggregate size (P<0.0001). Specifically, treatment with Dex, MEKi, and GA resulted in tighter and more compact sheets than treatment with control vehicles H_2_O and DMSO (P<0.001). Dex appeared to be more effective in promoting compaction than the other 2 agents (P<0.001). In contrast, treatment with MEKi and GA resulted in similar compaction profiles (P>0.05). Interestingly, aggregate compaction appears to correlate with the amount of fibronectin matrix. Aggregates in which matrix was significantly upregulated, such as those treated with Dex or GA, compacted to a greater degree than those in which matrix was reduced, such as those treated with MEKi or carrier controls. Of note, is that drug treatment and restoration of FNMA did not involve upregulation of α5β1 integrin or increased FN secretion, since integrin expression and secretion appeared to be unaffected as determined by immunoblot analysis (Fig. 5C). Moreover, the drug-mediated increase in compaction does not appear to involve increased cadherin expression, since immunoblot analysis using a pan-cadherin antibody showed that cadherin levels were also unaffected by drug treatment (Fig. 5C). We also performed assays to determine whether drug treatment altered cell migration as this could influence spreading velocity. We treated cells with either carrier or drugs and performed migration assays as described in Fig. S3. No difference was observed between carrier-only controls and cells treated with either Dex or MEKi. We did observe a significant decrease in migration of GA-treated cells (Fig. S3). However, we also noted that GA-treatment substantially increased cell size from 16 µm to nearly 30 µm, making it more difficult for cells to squeeze through the 8 µm pores of the filter chamber within the time-frame of the experiment. This change in cell size could, in principle, explain why fewer GA-treated cells migrated through the filter. Accordingly, it is unlikely that drug treatment reduced dispersal velocity by influencing cell migration, but rather, that drug-treatment inhibited the ability of cells to detach from the mass.

### Drug treatment significantly reduces spreading velocity

**Figure 6:**
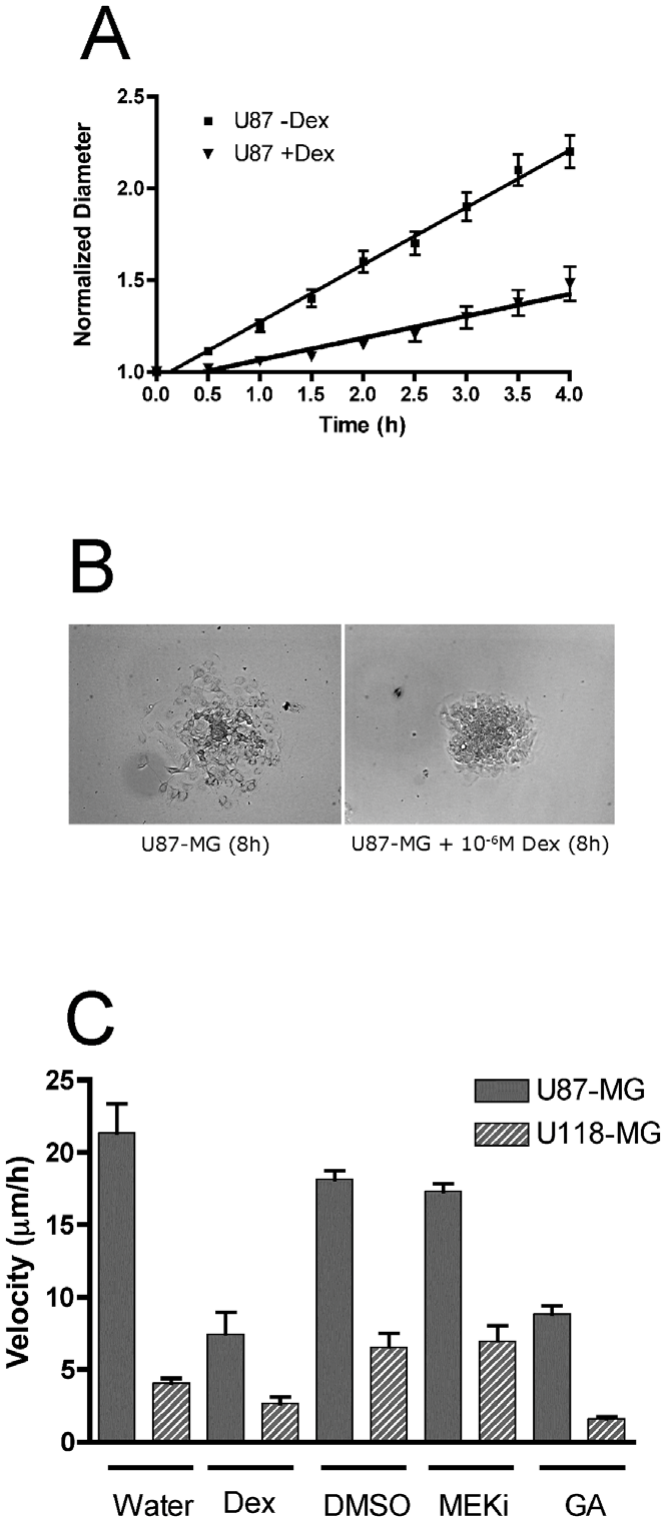
Effects of drug treatment on aggregate spreading velocity. A) Dex treatment significantly reduces the slope of the regression line. Aggregate spreading assays were performed using untreated and Dex-treated aggregates of U87-MG cells. Slopes were found to be significantly different (F-test, P<0.0001), with untreated aggregates having a steeper slope than that of their Dex-treated counterparts. B) Dex treatment promotes cell-cell contact during aggregate spreading. Depicted are phase-contrast images of control and Dex-treated aggregates of U87-MG glioma cells after 8 hours of incubation. Dex treatment markedly reduces spreading and appears to promote cell-cell cohesion at the advancing cell front. C) Spreading velocity of control and drug-treated aggregates. Spreading velocity of aggregates of U87-MG (solid bars) and U118-MG (hatched bars) was compared in response to treatment with Dex, MEKi or GA. Treatment by Dex or GA consistently reduced aggregate dispersal velocity in both cell lines.

The Dex-mediated upregulation of FNMA observed for highly dispersive U87-MG cells significantly reduced the slope of the regression line. Fig. 6A shows that Dex-treatment reduced the slope from 0.309±0.007 to 0.1198±0.0104 and that the difference in slope was significant (F test, P<0.0001). The correlation coefficient, r^2^, for the regression lines was 0.99 and 0.95 for untreated and Dex-treated aggregates, respectively, demonstrating a direct linear relationship between normalized diameter and time. This was confirmed by Run's test, which showed that the regression line was not significantly non-linear (P<0.0714). A reduction in the slope of the regression line signifies a decrease in aggregate spreading velocity. This decrease in spreading velocity is associated with an increase in cell-cell contact between cells dispersing from the aggregate mass. Fig. 6B shows that after 8-hours in culture, cells at the dispersal margin tend to maintain little contact as they disperse, whereas those of aggregates treated with Dex maintain contact as they spread. Movies of the spreading process with and without Dex can be viewed in Movies S1 and S2, respectively. As can be seen, untreated aggregates of U87-MG tend to disperse as isolated cells (Movie S1), whereas Dex-treated aggregates appear to spread out as an interconnected sheet (Movie S2).

Since Dex is known to have pleiotropic effects, other agents known to upregulate FNMA were assessed for their ability to reduce spreading velocity. As shown in Fig. 6C, Dex significantly reduced the spreading velocity of U87-MG cells from 21.4±2.0 µm/h to 7.5±1.4 µm/h (Tukey's MCT, P<0.001). GA had a similar effect, reducing spreading velocity from 18.2±0.6 µm/h for DMSO controls to 8.8±0.6 µm/h (Tukey's MCT, P<0.001). In contrast, treatment with MEKi did not significantly reduce velocity (Tukey's MCT, P>0.05). A similar pattern was observed for U188-MG aggregates. Here, GA also significantly reduced spreading velocity (Tukey's MCT, P<0.05), whereas MEKi appeared to have no effect (Tukey's MCT, P>0.05). Dex treatment also did not appear to significantly reduce aggregate spreading velocity when the data were compared by ANOVA and Tukey's MCT. However, when the spreading velocity of Dex-treated aggregates was compared only to the H_2_O carrier control and analyzed by Student's t-test, the difference in spreading velocity was significant (P = 0.015). Strictly speaking, while analysis by ANOVA/Tukey's MCT is statistically more robust, results of the t-test analysis better reflects direct observation and, therefore, may be more biologically relevant.

### Effects of the 70 KDa fibronectin fragment on matrix assembly and aggregate compaction

**Figure 7:**
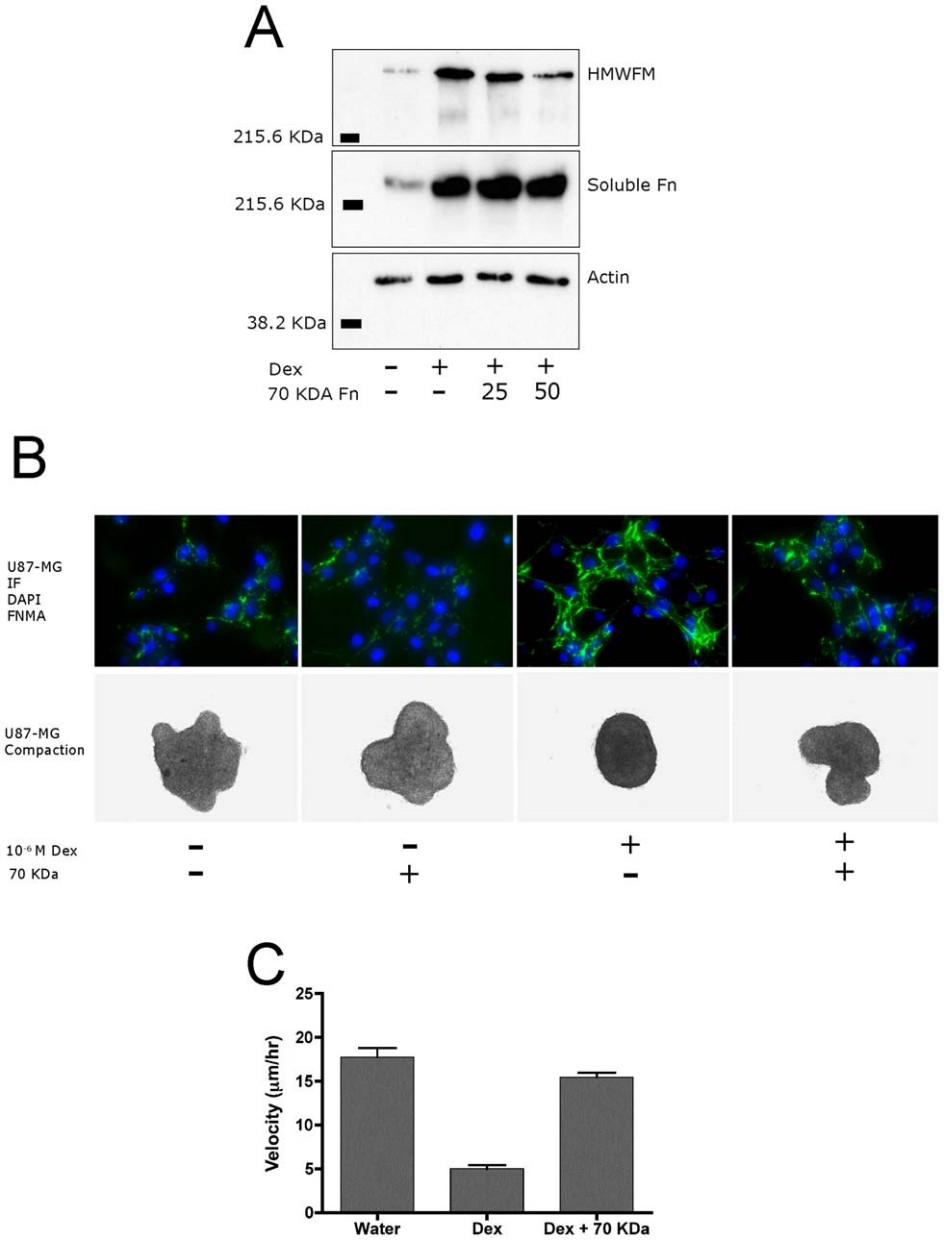
Blocking Dex-mediated FNMA rescues spreading velocity. A) Blocking of FNMA by the 70 KDa fragment of fibronectin. Aggregates of U87-MG cells generated and incubated in the absence and/or presence of the 70 KDa fragment were assessed for FNMA by differential solubilization assay. DOC-soluble and insoluble fractions were separated by SDS-PAGE and analyzed by immunoblotting using an anti-fibronectin antibody. Actin in the soluble fraction was used as loading control. As expected, Dex-treatment significantly increased the amount of soluble fibronectin and high molecular weight fibronectin multimers (HMWFM). Addition of the 70 KDa fragment had no effect on soluble fibronectin but significantly reduced the amount of insoluble FN matrix, represented here by a specific band of molecular weight >220 KDa. Knock-down of the FN matrix was more pronounced in aggregates generated and incubated in 50 µg/ml of the 70 KDa fragment. B) Blocking Dex-induced matrix assembly influences aggregate compaction. U87-MG cells were plated either in conventional 2D culture (IF panel) or in 3D hanging drop culture (compaction panel) in the absence and/or presence of Dex and 70 KDa fragment. Cells in 2D culture were incubated in anti-fibronectin antibody followed by an Alexa-488-conjugated secondary antibody. Fibronectin matrix is depicted in green. DAPI was used to label nuclei, here depicted in blue. Inclusion of the 70 KDa fragment in both untreated and Dex-treated aggregates alters the appearance of the matrix from one in which long fibers extend between cells to a more punctate pattern. A corresponding change in compaction was also noted; Dex-treated cells tending to form less compact aggregates when incubated in the presence of the 70 KDa fragment. C) Blocking Dex-induced fibronectin matrix assembly rescues aggregate spreading velocity. The spreading velocity of aggregates prepared in the presence of a combination of Dex and the 70 KDa fragment was greater than that of aggregates generated in Dex-containing medium alone and approached the spreading velocity of untreated aggregates.

To establish a mechanism of action of FNMA, we blocked matrix assembly after drug treatment and explored effects on spreading velocity of aggregates of U87-MG cells. Aggregates were generated in the presence of a 70 KDa fragment of fibronectin, which has previously been shown to block FN assembly [36]. The amount of 70 KDa fragment required to block matrix formation was determined by generating aggregates in the presence of either 25 µg/ml or 50 µg/ml 70 KDa and assessing effects on matrix assembly by immunoblot analysis. Fig. 7A shows that Dex treatment increases the amount of insoluble matrix detected and that the presence of the 70 KDa fragment significantly reduces matrix in a dose-dependent manner. Presence of the 70 KDa fragment had no effect on the amount of soluble fibronectin. Similar results were obtained when FNMA was assessed by immunofluorescence. Fig. 7B shows that addition of 50 µg/ml of 70 KDa fragment to both untreated and Dex-treated cells results in a reduced, more punctate, matrix as compared to cells incubated in absence of the blocking agent. Results of aggregate compaction assays demonstrate that Dex-treated aggregates compacted into tight spheres, whereas inclusion of the 70 KDa fragment gave rise to flat sheets of cells. As expected, addition of the 70 KDa fragment to untreated U87-MG cells had no effect. Having demonstrated that the 70 KDa fragment can block Dex-mediated matrix assembly and aggregate compaction, we explored effects on spreading velocity.

### Blocking FNMA rescues spreading velocity

Spreading velocity was assessed for aggregates that were either untreated, or treated with Dex and incubated in either the absence or presence of 50 µg/ml of the 70 KDa fragment. Fig. 7C shows that Dex-treatment, as expected, reduces spreading velocity from 17.8±1.0 µm/h to 5.02±0.4 µm/h (Tukey's MCT, P<0.001), but that inclusion of the 70 KDa fragment rescues the spreading velocity to 15.5±0.5 µm/h, a level approximating that of untreated aggregates (Tukey's MCT, P>0.05). These data support a direct role for FNMA during aggregate dispersal.

## Discussion

### The aggregate dispersal velocity assay

Spheroid formation by U87-MG, LN-229, and U118-MG is well-documented [21], [37] and can be best described as a behavior typical of viscoelastic liquids [38]. This liquid-like behavior can be exploited to measure key properties that have previously been shown to significantly influence tumor invasion. One such property, tumor surface tension, has been shown to be inversely proportional to invasive capacity [19], [21], [39]. Another such property, tumor dispersal velocity, was measured here. To measure dispersal velocity we assessed the rate of spreading, or “wetting”, of a solid substrate by a liquid aggregate. Adhesive forces between a liquid and solid cause a liquid drop to spread across the surface. Cohesive forces within the liquid cause the drop to ball up and avoid contact with the surface [30]. In cellular aggregates, these forces are represented by cell-ECM adhesion- and cell-cell cohesion mechanisms, respectively. In order to disperse, tumor cells must overcome forces holding them to the tumor mass and must also acquire sufficient traction to the substrate onto which they disperse. We plated aggregates composed of malignant glioblastoma cells onto tissue culture plastic in complete medium and observed that within 8 hours, aggregates had nearly spread to a monolayer. The spreading was linear and size-independent allowing us to directly measure spreading velocity.

### Dispersal velocity of three glioblastoma cell lines

The cell lines used in this study have been extensively characterized and used for decades. All were derived from tumors that originally had been classified as astrocytoma grade IV/glioblastoma [40]. Despite the similarity in pathologic grade, these three lines have been shown to possess different biomechanical properties that can markedly influence their invasive behavior. For example, aggregates of U87-MG cells were found to be less cohesive than those of LN-229 or U118-MG and that cohesion is inversely proportional to their invasive capacity [41]. In this study we showed that dispersal velocity also differed markedly; aggregates of U87-MG cells dispersing much more quickly than those of LN-229 and U118-MG. Interestingly, dispersal velocity appears to be inversely proportional to aggregate cohesion, consistent with the concept that it may be possible to decrease brain tumor dispersal by increasing the overall cell-cell cohesion of a tumor, effectively making it more difficult for cells to escape the tumor mass. Since dispersal is a hallmark of glioma, reducing dispersal could be of therapeutic benefit. Understanding the molecular mechanisms underlying tumor dispersal is fundamental to devising effective therapeutic strategies.

### Molecular determinants of glioblastoma dispersal

The difference in dispersal velocity between glioblastoma cells suggests that differences may exist in the expression or function of molecules controlling cell-cell cohesion or cell-ECM adhesion. In this study, we explored whether differences in cadherin expression could potentially explain differences in dispersal velocity. Immunoblot analysis using a pan-cadherin antibody failed to reveal any differences in cadherin expression between lines. Interestingly, a marked difference in integrin function *vis a vis* FNMA was observed. The role of integrins in gliomas is undisputed and integrins are currently emerging as a promising target of anticancer therapy [42]. Integrins most notably associated with glioma invasion are β1 integrin, which can then pair with several α subunits including α2, α3, α5, and α6 [43]. αvβ3 and αvβ5 are also over-expressed on both glioma cells and their vasculature [42]. The role most ascribed to integrins in gliomas is one of facilitating cell motility and migration. This study describes a different role, one in which α5β1 integrin function acts to discourage cell migration by increasing the overall cohesion of the tumor mass. In this view, α5β1 integrin–fibronectin interaction acts as a cellular glue, effectively connecting cells in 3D aggregates to one-another, thus discouraging detachment. We have previously reported that integrin-mediated cohesion is fibronectin-dependent, but more critically, that the fibronectin must be assembled into a fibrous matrix in order for aggregates to fully express their potential cohesive intensity [28]. Moreover, recent studies revealed that both integrin expression levels and the amount of available fibronectin must be optimized to fully support formation of a fibronectin matrix [44]. Thus, differences in α5 integrin expression or in fibronectin secretion could be responsible for differential capacity for FNMA between these three cell lines. This, however, is not the case since these lines appear to 1) express similar levels of surface α5 integrin as assessed by flow cytometry, and 2) secrete similar levels of fibronectin, as assessed by immunoblot analysis (data not shown). Therefore, the differences in the capacity for FNMA in the absence of differences in α5 integrin expression or fibronectin secretion by these cell lines, suggests a possible defect in α5β1 integrin function. Gliomas are known to exhibit high Ras activity [34] leading to constitutively active ERK, which has been shown previously to impede FNMA [35]. Inhibition of ERK MAP kinase activation by Dex, a synthetic glucocorticoid steroid commonly used to treat brain tumor related intracranial edema, enhanced FNMA in deficient cell lines [35]. We had previously shown that Dex treatment of U87-MG cells resulted in increased cohesion as measured by TST and that this was correlated with a marked decrease in invasive potential [21]. We therefore asked whether this increase in cohesion was due to restored capacity for FNMA and whether this would have an effect on dispersal velocity.

### Restoring FNMA significantly reduces aggregate dispersal velocity of glioma cells

We treated cells with Dex, MEKi, or GA, agents previously demonstrated to reactivate FNMA in cells defective in this process. Treatment with any of these agents restored FNMA by glioma cells and promoted aggregate compaction but not all to the same extent. MEKi, for example, stimulated FNMA to a lesser extent in both U87-MG and U118-MG, whereas Dex and GA had a greater effect. This was directly reflected in their spreading velocities. The effects of the drugs were more pronounced in highly dispersive U87-MG cells than in U118-MG, suggesting that restoration of FNMA may be more efficacious in controlling dispersal velocities of more highly aggressive cell lines or tumors. Restoration of FNMA was independent of de novo expression of α5 integrin or of increased fibronectin secretion, suggesting that treatment resulted in restoration of α5 integrin function rather than by genetically modifying expression of the molecules controlling the process. Previous studies from our laboratory have shown that cadherin expression is directly correlated with aggregate cohesion [45] and that this, in turn, is inversely proportional to aggregate spreading rate [33]. Accordingly, we also explored the possibility that drug treatment, in addition to activating FNMA, may also have resulted in the upregulation of cadherin expression. This was not the case since cadherin expression appeared to be unaffected by drug treatment. Effectively, Dex treatment reduced spreading velocity by shifting the balance of forces mediating spreading to favor cell-cell cohesion. Restoring FNMA reduced dispersal velocity by increasing the overall cohesion of the tumor, thus reducing the detachment of cells from the tumor mass.

### Blocking Dex-induced FNMA with the 70 KDa fragment of fibronectin rescues dispersal velocity

In order to address whether the drug-induced increase in FNMA is functionally responsible for the observed decrease in aggregate spreading velocity, we performed assays in which FNMA was induced by Dex treatment. We then measured spreading velocity in the absence or presence of the amino-terminal 70 KDa fragment of fibronectin. Because this fragment binds reversibly to cell surfaces with the same affinity as the native protein, it blocks incorporation of fibronectin into a fibrous matrix [36]. In principle, incubating aggregates in Dex and in 70 KDa fragment, should block matrix assembly and thus rescue spreading velocity. We show that U87-MG cell aggregates generated in the presence of Dex and 50 µg/ml 70 KDa fragment exhibit a markedly reduced capacity to assemble fibronectin and that whatever matrix did assemble did so in a punctate pattern rather than as fibers. This apparent reduction in FNMA resulted in a significant rescue of dispersal velocity similar to that of untreated controls. Collectively, these data provide evidence that differences in dispersal velocity or aggregate cohesion between glioblastoma cell lines is their capacity for FNMA. To our knowledge, this is the first demonstration that α5β1 integrin-fibronectin interaction can function as a dispersal suppressor in glioblastoma cells.

Whether FNMA plays a role clinically has yet to be determined. One recent study using human brain tumors and an animal model of glioma invasion showed that down-regulation of FNMA can render glioma cells more resistant to chemotherapy [46]. Our study suggests that increasing FNMA may in fact be protective by discouraging cell detachment from the tumor mass. These two points of view are not incompatible. There may in fact be an optimum level of FNMA that could still sensitize cells to chemotherapy while also discouraging escape of those cells from the tumor mass. Clearly no single factor can be predictive of clinical outcome. However, it is equally clear that dispersal of tumor cells is fundamentally linked to a poor prognosis. We showed that despite the fact that these cell lines were derived from tumors that were all staged as GBM, they possess distinctly different dispersal velocities that appear to correlate with their ability to assemble fibronectin into a matrix. Tumors composed of cells that possess this ability and are therefore more cohesive, would disperse more slowly or to a lesser degree. Since dispersal is a hallmark of gliomas, these differences could potentially explain why some patients diagnosed with glioblastoma may live for years, rather than months. It is also possible that the observed differences in dispersal velocity between these cell lines are purely artifact of tissue culture and that such differences do not exist in human brain tumors. To address this question would require a comprehensive assessment of FNMA in human brain tumor samples or in freshly isolated primary cell lines, followed by measurements of dispersal velocity, and ultimately, connection of this and other parameters, to clinical outcome We showed that Dex and other agents that upregulate matrix assembly can reduce dispersal velocity. While Dex is routinely used to treat brain tumor-related intracranial edema, we do not suggest that Dex or the other drugs specifically used in this study are of clinical relevance as potential therapy. Rather, we suggest that conceptually, the dispersal velocity assay may represent a platform for testing agents aimed at reducing dispersal by increasing aggregate cohesion, whether by up-regulating FNMA or by some other mechanism. An approach to limit dispersal and elucidation of the molecular determinants underlying the process are of fundamental importance to improve clinical outcome.

## Supporting Information

Figure S1 **Spreading velocity is independent of aggregate size.** We tested whether aggregate size could influence spreading velocity by pooling all data and comparing the diameter of each aggregate to its matched spreading velocity. The line describing this relationship has a slope of −0.021. An F-test revealed that the slope of the line is not significantly non-zero (P<0.554). These data indicate that velocity is independent of aggregate size.
(TIF)

Figure S2 **Transfilter migration of U87-MG, LN-229 and U118-MG cells.** We compared the migration rates of the three cell lines by plating 1×10^4^ cells into the top of an 8 µm transfilter chamber and counting the number of cells that migrated to the underside of the filter after overnight culture. Cells were plated onto 2 filters for each cell line. Cells remaining in the upper chamber were wiped off using a cotton swab. Those on the bottom aspect of the filter were stained using SYTO-16 nuclear stain. Four images, representing almost all of the filter surface area, were collected for each filter. Images of the cell nuclei were captured and images were analyzed using ImageJ. The number of migrating cells/field were compared by ANOVA and Tukey's MCT. No difference was detected (p>0.05).
(TIF)

Figure S3 **Migration of U87-MG cells in response to drug-treatment.** We compared the transfilter migration rates of untreated and drug-treated U87-MG cells to determine whether drug treatment significantly influenced cell motility. Analysis of the data by ANOVA and Tukey's MCT revealed that Dex and MEKi treatment did not significantly alter migration (p>0.05). However, GA appeared to markedly reduce the number of cells that were able to migrate through the 8 µm pores of the filter. We also noted that treatment by GA significantly increased cell size from an average of 17 µm for the carrier control and Dex or MEKi-treated cells to approximately 30 µm. This increase in size could, in principle, significantly impact the ability of cells to migrate through the 8 µm pores of the trans-well filter.
(TIF)

Movie S1 **Time-lapse movie of untreated aggregates of U87-MG cells.** Aggregates were plated on tissue culture plastic in complete CO_2_-independent medium and incubated on a heated stage of an inverted microscope. Images were captured every five-minutes for 8-hours. A time-lapse movie of aggregate spreading was generated by importing the Tiff images into Quicktime. Untreated aggregates of U87-MG tend to spread as single cells.
(MOV)

Movie S2 **Time-lapse movie of Dex-treated aggregates of U87-MG cells.** Cells were treated with 10^−6^ M Dex for 16 hours and aggregates were generated as previously described. Aggregates were plated on tissue culture plastic in complete CO_2_-independent medium containing 10^−6^ M Dex, and incubated on a heated stage of an inverted microscope. Images were captured every five-minutes for 8-hours. A time-lapse movie of aggregate spreading was generated by importing the Tiff images into Quicktime. Dex-treatment appears to increase cell-cell cohesion at the advancing cell front and aggregates appear to spread as sheets.
(MOV)
