# Supplementary figures and images for: Fibronectin Matrix Assembly Suppresses Dispersal of Glioblastoma Cells
Source: PLoS One. 2011 Sep 30;6(9):e24810. doi: 10.1371/journal.pone.0024810 (PMC3184095; doi:10.1371/journal.pone.0024810)

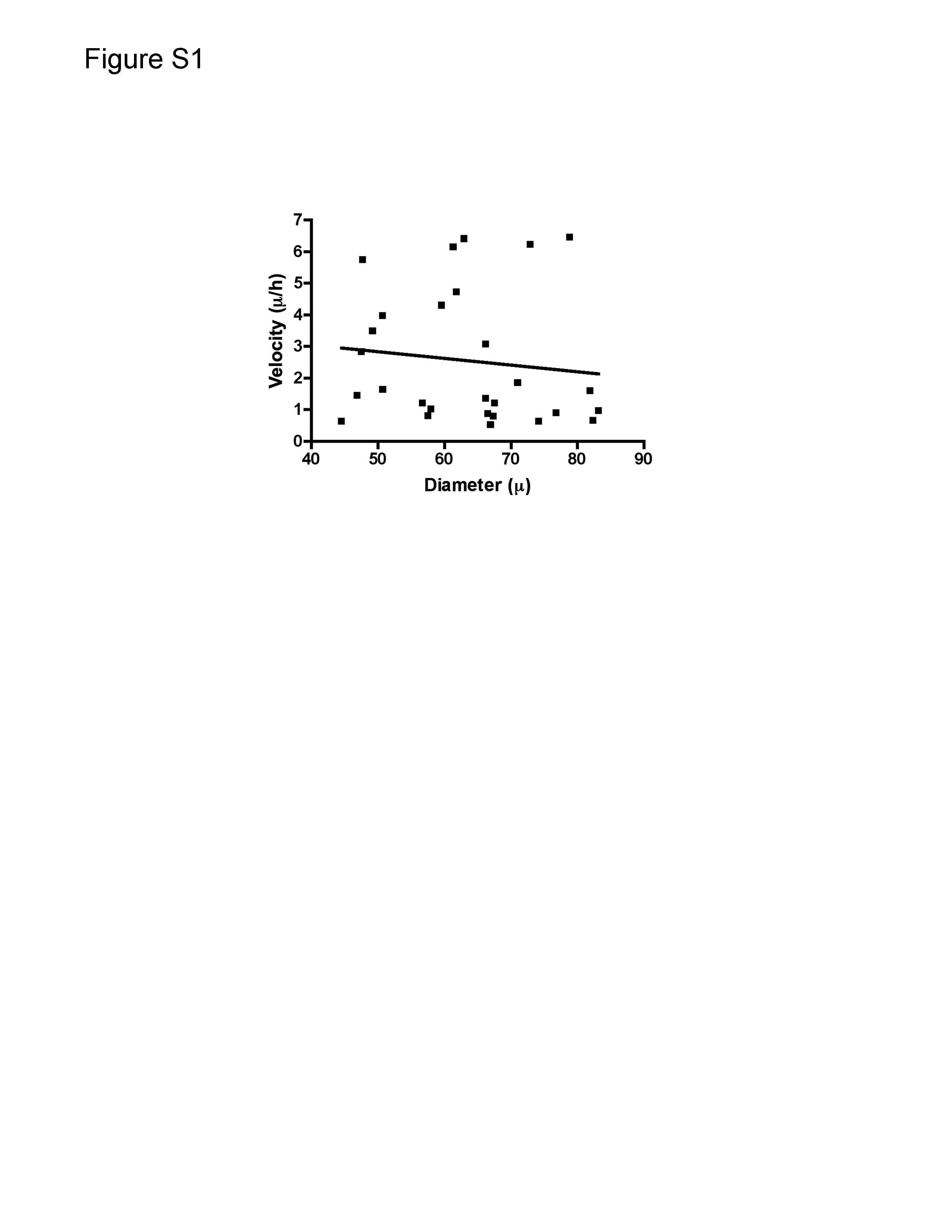

Supplement: Figure S1 — Spreading velocity is independent of aggregate size. We tested whether aggregate size could influence spreading velocity by pooling all data and comparing the diameter of each aggregate to its matched spreading velocity. The line describing this relationship has a slope of −0.021. An F-test revealed that the slope of the line is not significantly non-zero (P<0.554). These data indicate that velocity is independent of aggregate size. (TIF) [file pone.0024810.s001.tif]

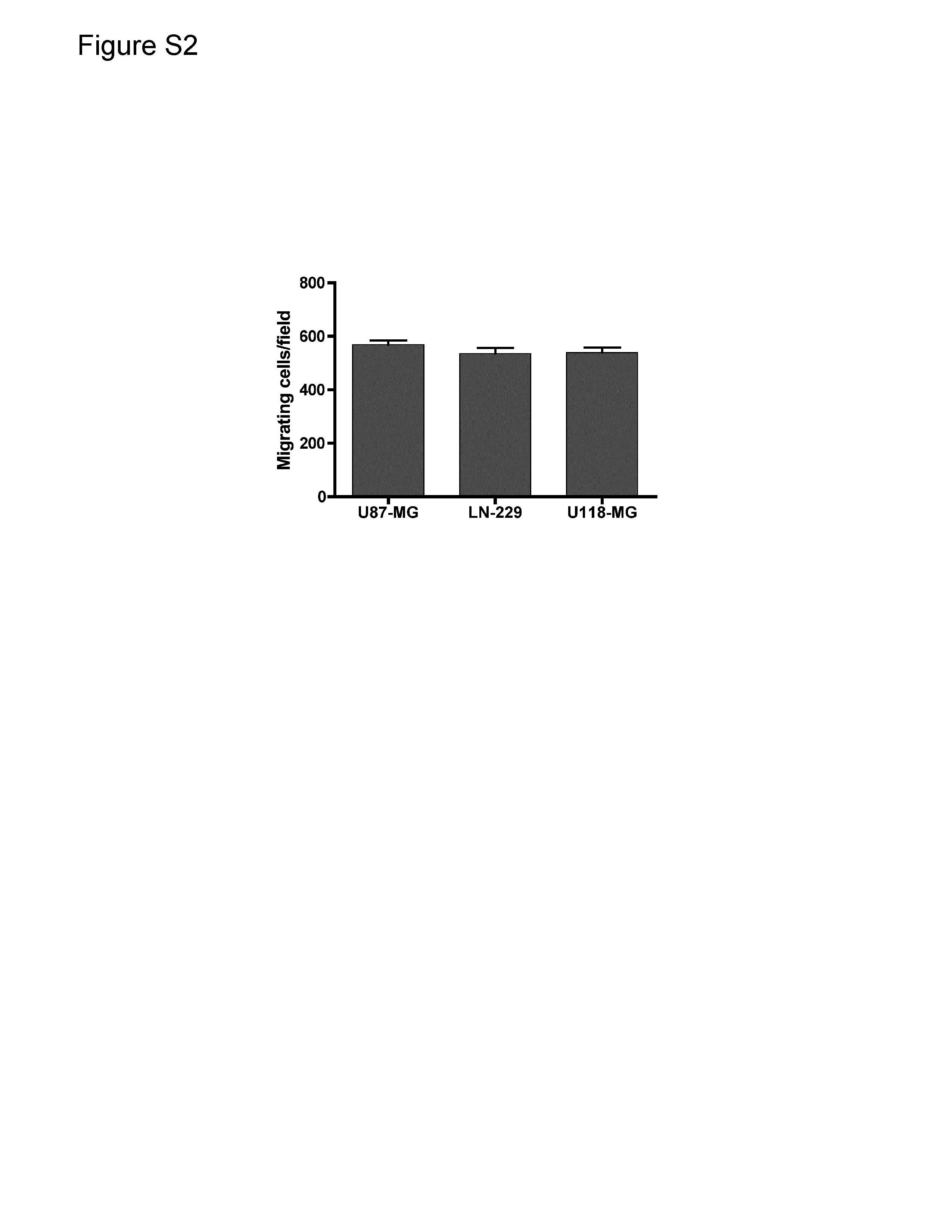

Supplement: Figure S2 — Transfilter migration of U87-MG, LN-229 and U118-MG cells. We compared the migration rates of the three cell lines by plating 1×10^4^ cells into the top of an 8 µm transfilter chamber and counting the number of cells that migrated to the underside of the filter after overnight culture. Cells were plated onto 2 filters for each cell line. Cells remaining in the upper chamber were wiped off using a cotton swab. Those on the bottom aspect of the filter were stained using SYTO-16 nuclear stain. Four images, representing almost all of the filter surface area, were collected for each filter. Images of the cell nuclei were captured and images were analyzed using ImageJ. The number of migrating cells/field were compared by ANOVA and Tukey's MCT. No difference was detected (p>0.05). (TIF) [file pone.0024810.s002.tif]

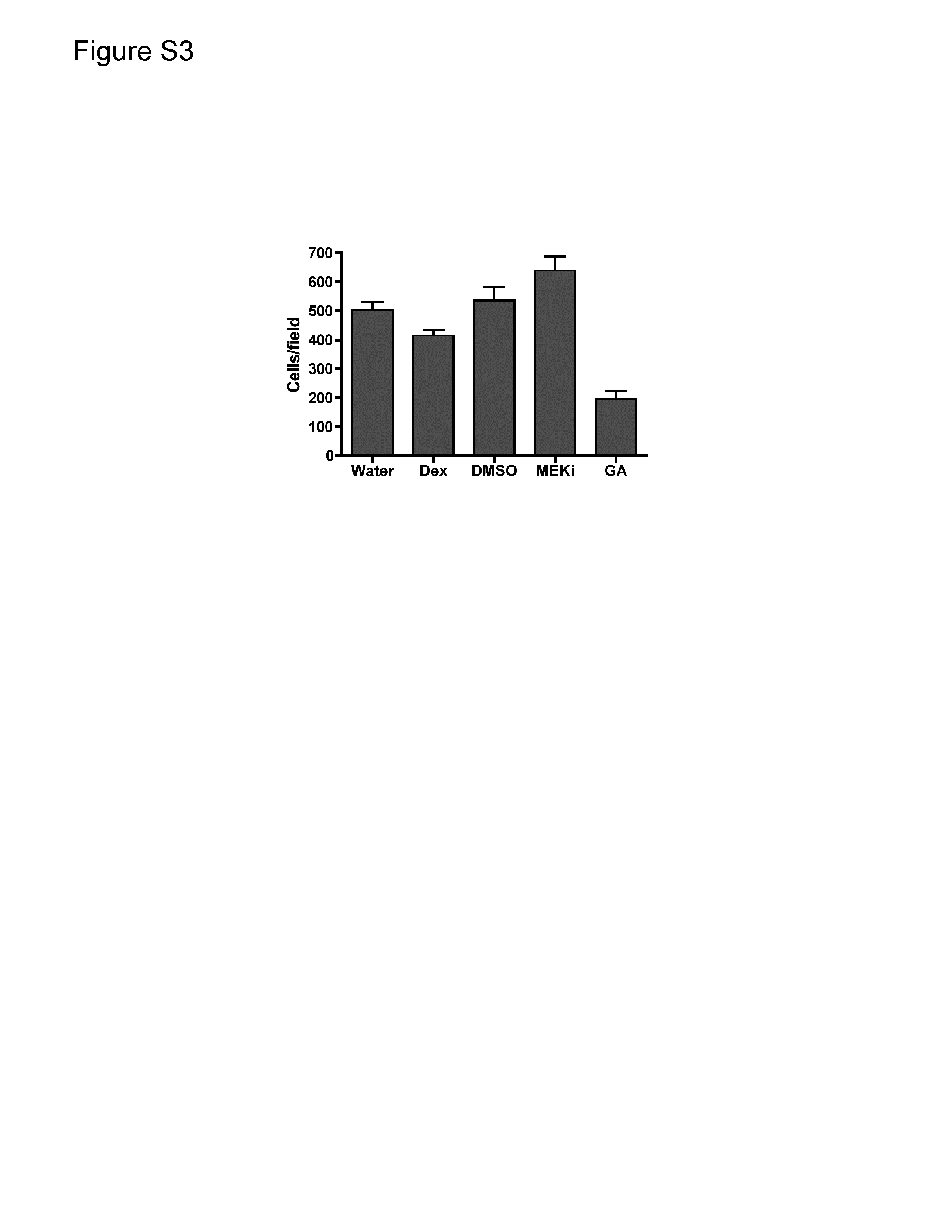

Supplement: Figure S3 — Migration of U87-MG cells in response to drug-treatment. We compared the transfilter migration rates of untreated and drug-treated U87-MG cells to determine whether drug treatment significantly influenced cell motility. Analysis of the data by ANOVA and Tukey's MCT revealed that Dex and MEKi treatment did not significantly alter migration (p>0.05). However, GA appeared to markedly reduce the number of cells that were able to migrate through the 8 µm pores of the filter. We also noted that treatment by GA significantly increased cell size from an average of 17 µm for the carrier control and Dex or MEKi-treated cells to approximately 30 µm. This increase in size could, in principle, significantly impact the ability of cells to migrate through the 8 µm pores of the trans-well filter. (TIF) [file pone.0024810.s003.tif]
